# Integrated Analyses of Gene Expression Profiles Digs out Common Markers for Rheumatic Diseases

**DOI:** 10.1371/journal.pone.0137522

**Published:** 2015-09-09

**Authors:** Lan Wang, Long-Fei Wu, Xin Lu, Xing-Bo Mo, Zai-Xiang Tang, Shu-Feng Lei, Fei-Yan Deng

**Affiliations:** 1 Center for Genetic Epidemiology and Genomics, School of Public Health, Soochow University, Suzhou, Jiangsu 215123, P. R. China; 2 Jiangsu Key Laboratory of Preventive and Translational Medicine for Geriatric Diseases, School of Public Health, Soochow University, Suzhou, Jiangsu 215123, P. R. China; University of Sydney, AUSTRALIA

## Abstract

**Objective:**

Rheumatic diseases have some common symptoms. Extensive gene expression studies, accumulated thus far, have successfully identified signature molecules for each rheumatic disease, individually. However, whether there exist shared factors across rheumatic diseases has yet to be tested.

**Methods:**

We collected and utilized 6 public microarray datasets covering 4 types of representative rheumatic diseases including rheumatoid arthritis, systemic lupus erythematosus, ankylosing spondylitis, and osteoarthritis. Then we detected overlaps of differentially expressed genes across datasets and performed a meta-analysis aiming at identifying common differentially expressed genes that discriminate between pathological cases and normal controls. To further gain insights into the functions of the identified common differentially expressed genes, we conducted gene ontology enrichment analysis and protein-protein interaction analysis.

**Results:**

We identified a total of eight differentially expressed genes (TNFSF10, CX3CR1, LY96, TLR5, TXN, TIA1, PRKCH, PRF1), each associated with at least 3 of the 4 studied rheumatic diseases. Meta-analysis warranted the significance of the eight genes and highlighted the general significance of four genes (CX3CR1, LY96, TLR5, and PRF1). Protein-protein interaction and gene ontology enrichment analyses indicated that the eight genes interact with each other to exert functions related to immune response and immune regulation.

**Conclusion:**

The findings support that there exist common factors underlying rheumatic diseases. For rheumatoid arthritis, systemic lupus erythematosus, ankylosing spondylitis and osteoarthritis diseases, those common factors include TNFSF10, CX3CR1, LY96, TLR5, TXN, TIA1, PRKCH, and PRF1. In-depth studies on these common factors may provide keys to understanding the pathogenesis and developing intervention strategies for rheumatic diseases.

## Introduction

Rheumatic diseases are painful conditions usually characterized by inflammation, swelling, and pain in joints or muscles. More than 100 types of diseases are classified as rheumatic diseases, including many types of arthritis. The pathologic mechanisms of rheumatic diseases are complex, under the influence of both genetic and environmental factors. Although the rheumatic diseases have been studied for decades, the underlying mechanisms are still poorly understood.

Rheumatic diseases share some common symptoms, which imply that they may share common pathologic factors. Though each rheumatic disease has specific genetic causes, previous studies identified common factors associated with different rheumatic diseases. For instance, the HLA region is known to be associated with several rheumatic diseases including rheumatoid arthritis (RA)[1], systemic lupus erythematosus (SLE)[2], ankylosing spondylitis (AS) [3], Sjögren's syndrome, as well as many others [4]. Tumour necrosis factor has been shown to play a dominant role in the pathogenesis of various immune-mediated inflammatory diseases such as RA, AS, osteoarthritis (OA) [5], and psoriatic arthritis [6]. In addition, hypovitaminosis D is commonly observed in rheumatic patients with and without autoimmune features [7]. Despite the above sporadic evidence, common factors shared by various rheumatic diseases have yet to be examined systematically.

Gene expression profiling with microarray is a powerful tool for the discovery of genes and biological pathways that are associated with various complex diseases [8, 9]. Gene expression datasets, collected in rheumatic patients and normal controls, have been accumulated for a decade, leading to successful identification of gene expression signatures for each rheumatic disease, respectively. However, to the best of our knowledge, no study has been conducted to systematically identify factors in common for various rheumatic diseases.

In this study, based on the archived public high-throughput microarray gene expression datasets for rheumatic cohorts, we performed comprehensive statistical analyses to identify genetic factors commonly significant for different rheumatic diseases.

## Materials and Methods

### Ethics statement

The study did not made use of human or vertebrate animal subjects and tissue. The data were collected from an international publicly available database and analyzed anonymously. Therefore, no additional informed consent was required.

### Collection and selection of data

**Fig 1:**
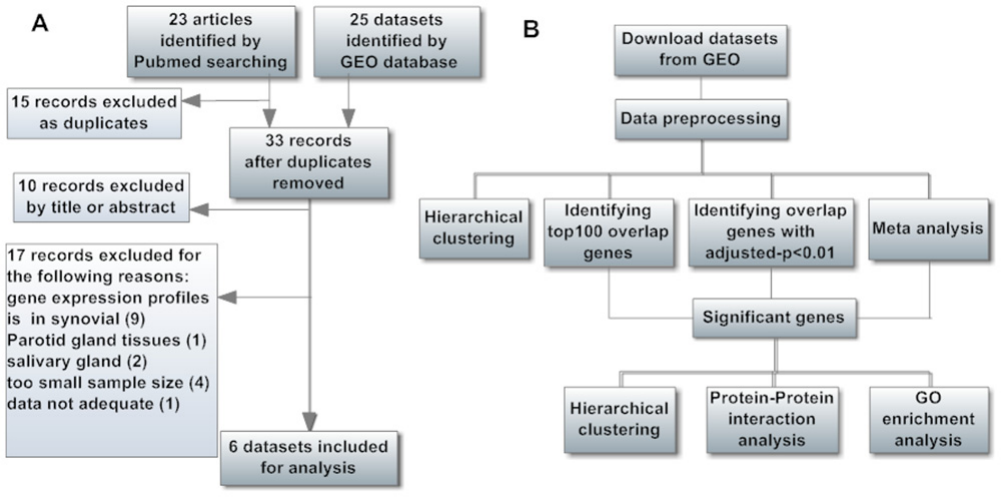
Flowcharts of Data Preparation and Data Analyses. (A) The selection process of microarray datasets. (B) The analysis process of the microarray datasets.

We searched the NCBI PubMed and Gene Expression Omnibus (GEO) database (http://www.ncbi.nlm.nih.gov/geo/)[10]with key words “Rheumatic diseases”, “Rheumatoid arthritis”, “Adult onset still’s disease”, “Osteoarthritis”, “Ankylosing spondylitis”, “Sjogren’s syndrome”, “Systemic lupus erythematosus”, “Microarray”, and “Gene expression profile”. By August 31, 2014, a total of 23 types of common rheumatic diseases were considered. A study was included in our analysis if: (1) it included patients diagnosed with rheumatic diseases and normal controls, (2) it contained gene expression profiling of blood, and (3) it provided data sufficient to our analysis. Finally, 6 datasets covering 4 types of rheumatic diseases(RA, SLE, OA, and AS) were retained for subsequent analysis (Table 1). The process of data collection and selection was provided in Fig 1A.

**Table 1:** Characteristics of the Datasets Included in the Analysis.

Disease Type	GEO Accession	Platform	Case	Control	Tissue
Rheumatoid arthritis (RA1)	GSE15573	GPL6102	18	15	PBMCs
Rheumatoid arthritis (RA2)	GSE1402	GPL8300	20	11	PBMCs
Systemic lupus erythematosus (SLE1)	GSE12374	GPL1291	11	6	PBMCs
Systemic lupus erythematosus (SLE2)	GSE20864	GPL1291	21	45	PBMCs
Osteoarthritis (OA)	GSE48556	GPL6947	106	33	PBMCs
Ankylosing spondylitis (AS)	GSE25101	GPL6947	16	16	Whole blood

PBMCs: peripheral blood mononuclear cells.

### Data preprocessing

We downloaded the 6 microarray gene expression datasets from GEO. The datasets were generated using four different platforms. The numbers of profiled genes in each dataset are different, and the probe IDs are different across platforms. To perform integrative analysis of datasets from different studies, we processed the datasets by using functions *MetaDE*.*match*, *MetaDE*.*merge*, *MetaDE*.*filtering* inan R package: MetaDE [11]. At the first step, when multiple probes matched to the same gene, we adopted the “IQR” method to select a probe with the largest interquartile range of gene expression values among all matched probes to represent the gene. At the second step, we extracted the commonly profiled genes across the six datasets. In the identification of differential expressed genes (DEGs), either un-expressed or un-informative genes contribute to false discoveries. Thus, at the third step, we performed gene filtering to sequentially remove un-expressed genes and un-informative genes. In each datasets, mean intensities and standard deviations of expression valuesfor each gene were ranked. The sum of ranks across all datasets was used to evaluate level of gene expression/information. To get the best balance between the false discovery rate (FDR)and the number of genes retained, we considered 30% genes with the smallest rank sum of mean intensity as un-expressed genes, and considered 30% genes with the smallest rank sum of standard deviations as un-informative genes [11]. Finally, a total of 2600 genes were retained for further analysis.

### Statistical analysis

We analyzed each dataset individually by *ind*.*analysis* function in MetaDE package to identify DEGs between rheumatic patients and normal controls. The moderatedt-statistic [12] was selected for significance analysis. The Benjamini & Hochberg FDR method was used to apply p-value adjustment for multiple-testing correction [13].

To get an overview of similarities of the gene expression profiles among different rheumatic diseases, we considered an approach introduced by Marina Sirota [14]. In a specific dataset, expression variation score for each gene is calculated as *sign*(*t*_*j*_) *log*(*p*_*j*_), where j is a gene number in a specific dataset and t_j_ is the moderated-t statistic of gene j, p_j_ is the moderatep-value of gene j. Thus it combines both the strength and the ‘direction’ of association. The gene expression variation profile of each dataset is a set of expression variation scores of all genes in the dataset. Correlations between the gene expression variation profiles can quantify the similarity of gene expression and regulation effects [14]. Herein, we considered the Kendall and Spearman correlation methods, as these two correlations are well-known methods for quantifying the degree of correlations between lists of ordinal data. The above correlation coefficients were computed by using the *cor*.*test* function in R. Based on the correlation coefficients, the hierarchical clustering of datasets was conducted by using the gplots package in R.

To investigate the common DEGs across the four types of rheumatic diseases, we firstly ranked the moderate p-values from the smallest to the largest for each dataset. Then, we examined the overlaps of the top 100 ranked genes across the six datasets. Genes with significantly differential expression in at least 3 types of rheumatic diseases were selected as common genes. Under the same criterion, we also detected common genes from DEGs with FDR adjusted p-value less than 0.01.

To evaluate the reliability of the above detected common markers, we performed a meta-analysis of the 6 gene expression microarray datasets. We used MetaDE package for identification of DEGs by the Fisher method and the maximum P-value method. The moderated t-statistic was used to calculate p-values in each datasets, and the Benjamini & Hochberg FDR method was used to apply meta-p-value adjustment. In the Fisher method, strong statistical significance of a gene could result from an extremely small p-value of one study, thus it detects genes that are differentially expressed in one or more datasets. In contrast, the maximum P-value method detects genes with small p-values of all studies [15].

### Functional annotation analysis

To understand the functions of the identified genes, we performed Gene Ontology (GO) enrichment analysis (http://www.geneontology.org/). Herein, we only considered the biological process. To explore the functional associations between identified genes, the proteins encoded by the identified genes were analyzed according to the Search Tool for the Retrieval of Interacting Genes/Proteins (STRING) 9.1 Server (http://www.string-db.org/) [16].

The above data processing and analyses workflow was presented in Fig 1B.

## Results

### Study characteristics

A total of 6 GEO datasets (accession number: GSE15573, GSE1402, GSE12374, GSE20864, GSE48556, and GSE25101) were included in our analysis, covering 4 types of rheumatic diseases (2, 2, 1, 1, datasets on RA, SLE, OA, and AS, respectively). These datasets were generated with total RNA extracted from peripheral blood of rheumatic patients and normal controls [17–22]. Detailed information of each dataset was described in Table 1. To be noted, four different microarray platforms were used in generating the six datasets (GPL6102 for GSE15573, GPL8300 for GSE1402, GPL1291 for GSE12374 and GSE20864, GPL6947 for GSE48556 and GSE25101). After preprocessing the datasets, a total of 2600 genes, profiled in all the 6 datasets, were extracted for analysis in the present study.

### Correlation of gene expression variation profiles between rheumatic diseases

**Fig 2:**
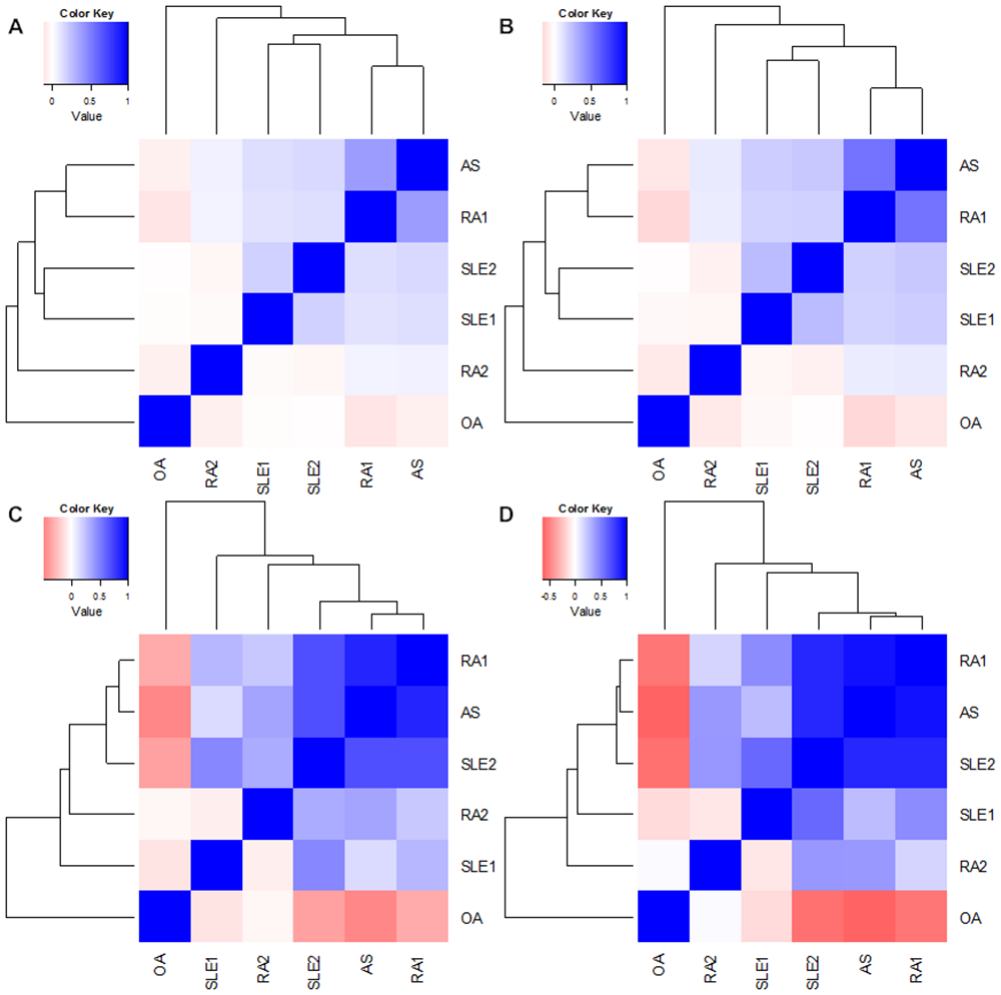
Disease Heatmap Based on Gene Expression Variation Profiles. This diagram shows correlations between gene expression variation profiles of various rheumatic diseases. (A) Hierarchical cluster with Kendall correlation based on the whole gene expression variation profile; (B) Hierarchical cluster with Spearman correlation based on the whole gene expression variation profile; (C) Hierarchical cluster with Kendall correlation based on the eight common genes; (D) Hierarchical cluster with Spearman correlation based on the eight common genes. Positive and negative correlations between pairs of diseases are shown in blue and pink, respectively.

The Kendall and Spearman correlation analyses, in Fig 2A and 2B respectively, presented similar results. Correlation matrices between the gene expression variation profilesof datasets were shown in the heat maps. Positive correlations between pairs of diseases were shown in blue, and negative correlations were shown in pink. The first level of clustering put RA1 and AS in the same cluster; SLE1 and SLE2 were put in the same cluster in the second level of clustering. The two clusters then converged and clustered together with RA2. However, OA was negatively correlated with the other five datasets, and did not cluster with others until at the last level of clustering. These results were consistent with previous studies [14, 23]that suggest similarities and differences between rheumatic diseases.

### Identification of common DEGs in rheumatic diseases

Based on the moderate p-values, top100 ranked genes in each datasets were listed in S1 Table. Genes with adjusted-p<0.01 in each dataset were listed in S2 Table. Throughout S1 and S2 Tables, none of the genes was identified in all the four studied rheumatic diseases. Corresponding to S1 and S2 Tables, genes identified in three of the four diseases were presented in Table 2. Each ranking method identified five genes. Notably, TXN and CX3CR1 were identified by both ranking methods. Together, a total of eight genes were identified, includingTNFSF10, CX3CR1, LY96, TLR5, TXN, TIA1, PRKCH, and PRF1. Significance of the above eight genes for rheumatic diseases was further evidenced by meta-analyses with the Fisher’s method (Table 2). Four of the DEGs were further validated by meta-analyses using the max-P method (Table 2).

**Table 2:** Differentially Expressed Genes Identified in Three Types of Studied Rheumatic Diseases.

Gene Symbol	P-value						Meta P-value	
	RA1	RA2	SLE1	SLE2	OA	AS	Fisher	Max-P
TNFSF10 ^a^	**1.80E-04**	**6.00E-04**	**9.00E-04**	**1.00E-20**	5.66E-01	**1.50E-03**	**1.07E-19**	1.65E-01
LY96 ^a^	**1.20E-04**	2.44E-02	4.49E-02	**1.00E-20**	1.90E-02	**7.95E-05**	**1.07E-19**	**2.74E-18**
PRKCH ^a^	**5.13E-06**	7.25E-02	2.52E-01	5.86E-02	**3.21E-05**	**5.10E-04**	**1.07E-19**	1.01E-02
TXN ^a^	**1.00E-20**	1.80E-02	1.79E-01	**1.00E-20**	6.51E-01	**1.28E-05**	**1.07E-19**	2.71E-01
CX3CR1 ^a^	7.07E-02	**2.00E-04**	3.90E-02	1.52E-02	**1.00E-20**	**3.46E-05**	**1.07E-19**	**2.74E-18**
TXN ^b^	**1.63E-18**	5.83E-02	7.20E-01	**1.61E-19**	7.79E-01	**7.33E-03**	**1.07E-19**	2.71E-01
CX3CR1 ^b^	2.58E-01	**6.76E-03**	5.83E-01	4.68E-02	**2.00E-18**	**8.18E-03**	**1.07E-19**	**2.74E-18**
TLR5 ^b^	4.34E-02	**6.76E-03**	3.77E-01	**6.94E-05**	**5.49E-03**	4.05E-01	**1.07E-19**	**2.28E-03**
TIA1 ^b^	3.49E-01	**6.76E-03**	7.36E-01	**1.09E-03**	**6.66E-03**	5.33E-01	**1.07E-19**	1.58E-02
PRF1 ^b^	6.26E-02	**8.82E-03**	7.08E-01	**6.60E-04**	**1.88E-03**	6.43E-02	**1.07E-19**	**4.76E-04**

Presented are p values from tests of differential expression between rheumatic patients and normal controls.

^a^ Genes identified from the top 100 ranked genes across the six datasets. The corresponding p-values are moderate p-values from the moderated-t statistic.

^b^ Genes identified based on p-values adjusted by the Benjamini-Hochberg method. The corresponding p-values are adjusted p-values.

Each of the genes was identified from three of the four diseases, and the p values of the corresponding datasets are in bold.

Significant p values in the meta-analysis are in bold.

To ascertain the importance of the above eight genes for rheumatic diseases, we conducted a clustering analysis based on the eight detected genes. Consistent with the clustering analysis based on whole gene expression variation profiles, clustering analysis based on the above eight genes (Fig 2C and 2D) successfully separated OA from the other three autoimmune rheumatic diseases. Although clusters within autoimmune rheumatic diseases changed slightly, the global trend was similar and the correlations within the autoimmune rheumatic diseases were enhanced. This finding indicates that the identified genes may play a principle role in the molecular pathological mechanism of these diseases.

### Functional annotation

**Fig 3:**
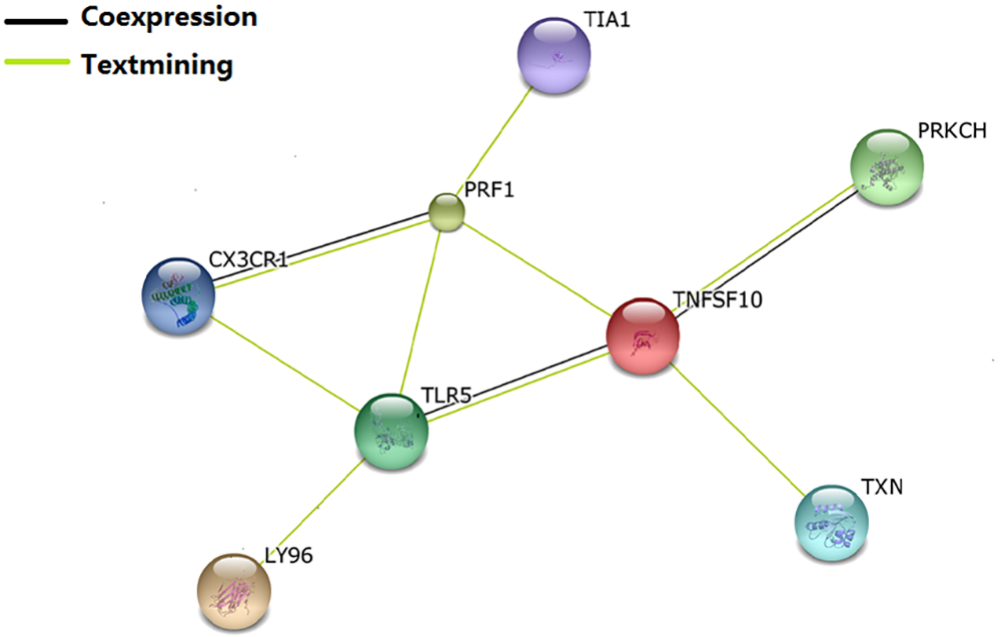
The Evidence View of Protein-Protein Interaction. The proteins were analyzed using the STRING database 9.1. The predicted functional interaction network is shown in the evidence view where the different line colors represent the types of evidence for the association.

PPI analysis of the eight identified genes provided reference information for their potential association (Fig 3). Each gene had text mining associations with others. In addition, there were co-expression relations between three pairs of genes: CX3CR1/PRF1, TLR5/TNFSF10, and TNFSF10/PRKCH. Four genes, i.e., CX3CR1, PRF1, TNFSF10, and TLR5, were the nodes of the network.

The top 20 enriched GO terms of biological process related to the above eight identified genes were shown in Table 3. The most significantly enriched function was “immune response” (GO:0006955, p = 6.70E-06). Related with immune regulation are GO terms “immune system process” (GO:0002376, p = 0.0001166) and “MyD88-dependent toll-like receptor signaling pathway” (GO:0002755, p = 0.004402). Notably, twelve of the 20 enriched GO terms (GO: 0006968, GO: 0071222, GO: 0071219, GO: 0071216, GO: 0006952, GO: 0051707, GO: 0043207, GO: 0009607, GO: 0032496, GO: 0002237, GO: 0071396, GO: 0009617) were related with biotic stimulus. The remaining GO terms were related with response to stress, signaling regulation, and cell communication.

**Table 3:** The Top 20 Significantly Enriched GO Terms of Biological Processes Involving the Eight Identified DEGs.

GO ID	Term	p-value	Genes
GO:0006955	immune response	6.70E-06	TNFSF10 LY96 TXN CX3CR1 TLR5 PRF1
GO:0006968	cellular defense response	1.39E-05	LY96 CX3CR1 PRF1
GO:0071222	cellular response to lipopolysaccharide	6.76E-05	LY96 CX3CR1 TLR5
GO:0071219	cellular response to molecule of bacterial origin	8.02E-05	LY96 CX3CR1 TLR5
GO:0002376	immune system process	1.17E-04	TNFSF10 LY96 TXN CX3CR1 TLR5 PRF1
GO:0071216	cellular response to biotic stimulus	1.24E-04	LY96 CX3CR1 TLR5
GO:0006952	defense response	3.68E-04	LY96 TXN CX3CR1 TLR5 PRF1
GO:0051707	response to other organism	5.11E-04	LY96 CX3CR1 TLR5 PRF1
GO:0043207	response to external biotic stimulus	5.11E-04	LY96 CX3CR1 TLR5 PRF1
GO:0009607	response to biotic stimulus	6.10E-04	LY96 CX3CR1 TLR5 PRF1
GO:0032496	response to lipopolysaccharide	6.47E-04	LY96 CX3CR1 TLR5
GO:0002237	response to molecule of bacterial origin	7.78E-04	LY96 CX3CR1 TLR5
GO:0071396	cellular response to lipid	1.44E-03	LY96 CX3CR1 TLR5
GO:0006950	response to stress	1.95E-03	LY96 TXN CX3CR1 TLR5 PRF1 PRKCH
GO:2001239	regulation of extrinsic apoptotic signaling pathway in absence of ligand	2.27E-03	TNFSF10 CX3CR1
GO:0009617	response to bacterium	3.44E-03	LY96 CX3CR1 TLR5
GO:0009967	positive regulation of signal transduction	3.47E-03	TNFSF10 LY96 TXN TLR5
GO:0023056	positive regulation of signal	4.14E-03	TNFSF10 LY96 TXN TLR5
GO:0010647	positive regulation of cell communication	4.23E-03	TNFSF10 LY96 TXN CX3CR1 TLR5
GO:0002755	MyD88-dependent toll-like receptor signaling pathway	4.40E-03	LY96 TLR5

## Discussion

The inflammation and abnormal immune process are two important pathologic characteristics for rheumatic diseases. Joint pain is the main symptom in common. We speculate that these diseases may share similar pathological factors and mechanisms. In the last decade, microarray analysis of gene expression profiles has been widely used to identify genes and biological pathways associated with various complex diseases [8, 9, 16, 18]. However, previous such studies on rheumatic diseases usually focused on identifying factors specific to one disease and paid little attention to identifying genes important to various diseases. Therefore, in this study, we are attempted to identify common genes underlying multiple rheumatic diseases, with RA, SLE, OA, and AS as representatives. To the best of our knowledge, this is the first such endeavor in the research community of rheumatic diseases.

By jointly analyzing 6 published microarray gene expression datasets about RA, SLE, OA and AS, we identified eight genes (TNFSF10, CX3CR1, LY96, TLR5, TXN, TIA1, PRKCH, PRF1) presenting general importance to rheumatic diseases. As evidenced by PPI and GO analyses, these eight genes interact with each other to exert functions related to immune response and immune regulation.

Consistent with our findings, evidences from previous studies support that four of the above eight identified genes, i.e., TNFSF10, CX3CR1, TLR5, and PRF1, are relevant to multiple rheumatic diseases. For example, it was reported that TNFSF10 is involved in pathogenesis of RA [24], SLE [25], AS [26], OA [27], and multiple sclerosis [28]. CX3CR1 was reported to involve in inflammation and autoimmune progresses in RA [29], MS [30] and SS [31]. In vivo experiments confirmed that the de novo CX3CL1-CX3CR1 axis plays a pivotal role in osteoclast recruitment and subsequent bone resorption [32], which provides a clue of molecular mechanism responsible for bone damage in rheumatic diseases. It is known that toll-like receptors (TLRs) are membrane receptors recognizing biotic inflammatory stimulus. Previous study showed that TLR5 was over-expressed in patients affected with AS [33], RA and OA [34]. Besides, TLR5 gene mutations are associated with resistance and susceptibility to SLE [35]. In addition, PRF1 was reported to be associated with SLE [36], RA [37] and AS Patients [38].

Besides the above four genes previously recognized to be involved in multiple rheumatic diseases, this study firstly point out that TXN, PRKCH, TIA1, and LY96 genes are significant for multiple rheumatic diseases, as well. Previous studies showed that TXN, PRKCH, and TIA1 genes are related to RA [39–41]. LY96 gene encodes a protein which associated with TLR4 on cell surface, and plays important roles in TLR signaling pathway, inflammatory response and innate immune response [42]. However, molecular function mechanisms of the above four genes in relation to other three types of rheumatic diseases are unclear, and have yet to be studied.

It iswell known that immune response is involved in the development of most rheumatic diseases. In this study, GO analyses showed that the eight identified genes are significantly enriched in biological processes of “immune response” and “defense response”. Specifically, those genes are involved in “responses to lipoplysaccharide, molecule of bacterial origin, biotic stimulus, and bacterium”, and are involved in “MyD88-dependent toll-like receptor signaling pathway”. The findings point out that gene-environment interaction plays an important role in the development of rheumatic diseases. Understanding the molecular functions of these genes in biotic induced inflammation and cellular defense responses may shed new light on the pathogenesis of rheumatic diseases.

The purpose of this study is to identify common genes for various rheumatic diseases. Due to the following reasons, this study has some limitations. Firstly, for limited availability of gene expression datasets, this study only focused on four kinds of rheumatic diseases. Datasets to be generated in the future for more kinds of rheumatic diseases may contribute to validating and identifying novel genes with rheumatic-diseases-general effects. Secondly, the gene expression datasets utilized in the present study were generated from four different microarray platforms. Only 2600 genes profiled in all the 6 datasets were analyzed in this study, leaving a majority of the protein-coding genes across the human genome unexplored yet. Thirdly, since the 6 utilized datasets were generated from blood samples, we are unable to identify the specific disease-related functional cells.

To understand which immune cell subsets are contributing to the whole blood expression of the identified genes, we searched the Immunological Genome (ImmGen) [43], a 'road map' of gene-expression in all immunecells. According to the ImmGen, PRF1, TNFSF10 and LY96 show relatively higher expression level in innate lymphocytes than other cell subsets. Besides, TNFSF10 is also highly expressed in stromal cells and gd T cells; LY96 is also highly expressed in stromal cells and macrophages. CX3CR1 is relatively highly expressed in macrophages, monocytes, and ab T cells. TIAL shows a moderately high expression level in stem cells, B cells, and T cells. PRKCH is relatively highly expressed in T cells. Other two genes, TLR5 and TXN, are not in the database. For our identified genes, the above expression patterns across various immune cell subsets provide us interesting clues to better understand and explore their functions in particular immunological cell subsets.

In conclusion, the present study identified eight common genes underlying different types of rheumatic diseases. In-depth functional studies on these common genes may improve our understanding of the pathological processes of these diseases, which could have important implications for the prevention and treatment of rheumatic diseases in general.

## Supporting Information

S1 Table The top 100 ranked genes that show a differential expression between rheumatic patients and normal controls. Ranking of genes is based on moderated p-values. Genes appeared in more than 2 types of rheumatic diseases are in red.
(XLSX)

S2 Table Genes with adjusted p-values <0.01 in each dataset. P-values are adjusted by the Benjamini-Hochberg method. Genes appeared in more than 2 types of rheumatic diseases are in red.
(XLSX)

## References

[1] Plenge RM, Seielstad M, Padyukov L, Lee AT, Remmers EF, Ding B, et al TRAF1-C5 as a risk locus for rheumatoid arthritis—a genomewide study. N Engl J Med. 2007;357(12):1199–209. 17804836 10.1056/NEJMoa073491 PMC2636867

[2] International Consortium for Systemic Lupus Erythematosus G, Harley JB, Alarcon-Riquelme ME, Criswell LA, Jacob CO, Kimberly RP, et al Genome-wide association scan in women with systemic lupus erythematosus identifies susceptibility variants in ITGAM, PXK, KIAA1542 and other loci. Nat Genet. 2008;40(2):204–10. 10.1038/ng.81 18204446 PMC3712260

[3] Tsui FW, Tsui HW, Akram A, Haroon N, Inman RD. The genetic basis of ankylosing spondylitis: new insights into disease pathogenesis. Appl Clin Genet. 2014;7:105–15. 10.2147/TACG.S37325 24971029 PMC4070859

[4] Goris A, Liston A. The immunogenetic architecture of autoimmune disease [review]. Cold Spring Harb Perspect Biol. 2012;4(3). 10.1101/cshperspect.a007260 PMC3282406 22383754

[5] He W, Pelletier JP, Martel-Pelletier J, Laufer S, Di Battista JA. Synthesis of interleukin 1beta, tumor necrosis factor-alpha, and interstitial collagenase (MMP-1) is eicosanoid dependent in human osteoarthritis synovial membrane explants: interactions with antiinflammatory cytokines. J Rheumatol. 2002;29(3):546–53. 11908571

[6] Blandizzi C, Gionchetti P, Armuzzi A, Caporali R, Chimenti S, Cimaz R, et al The role of tumour necrosis factor in the pathogenesis of immune-mediated diseases [review]. Int JImmunopathol Pharmacol. 2014;27(1 Suppl):1–10. 10.1177/03946320140270S101 24774503

[7] Bellan, Mattia, Sainaghi, Paolo P, Pirisi, Mario. Cholecalciferol High Dose Supplementation Should Be Preferred in Rheumatic Patients Independently to the Presence of an Autoimmune Disease. Arthritis Rheum. 2010;62 Suppl.

[8] Cooper-Knock J, Kirby J, Ferraiuolo L, Heath PR, Rattray M, Shaw PJ. Gene expression profiling in human neurodegenerative disease [review]. Nat Rev Neurol. 2012;8(9):518–30. 10.1038/nrneurol.2012.156 22890216

[9] Shi X, Shen S, Liu J, Huang J, Zhou Y, Ma S. Similarity of markers identified from cancer gene expression studies: observations from GEO. Brief Bioinform. 2014;15(5):671–84. 10.1093/bib/bbt044 23788798 PMC4271059

[10] Barrett T, Wilhite SE, Ledoux P, Evangelista C, Kim IF, Tomashevsky M, et al NCBI GEO: archive for functional genomics data sets—update. Nucleic Acids Res. 2013;41(Database issue):D991–5. 10.1093/nar/gks1193 23193258 PMC3531084

[11] Wang X, Kang DD, Shen K, Song C, Lu S, Chang LC, et al An R package suite for microarray meta-analysis in quality control, differentially expressed gene analysis and pathway enrichment detection. Bioinformatics. 2012;28(19):2534–6. 22863766 10.1093/bioinformatics/bts485 PMC3463115

[12] Smyth G, Gentleman R, Carey VJ, Huber W, Irizarry RA, Dudoit S. Bioinformatics and Computational Biology Solutions Using R and Bioconductor. Springer, New York 2005:pp.397–420.

[13] Benjamini Y, Hochberg Y. Controlling the false discovery rate: A practical and powerful approach to multiple testing. J. Roy. Statist. Soc. Ser. B. 1995;57:289–300.

[14] Sirota M, Schaub MA, Batzoglou S, Robinson WH, Butte AJ. Autoimmune disease classification by inverse association with SNP alleles. PLoS Genet. 2009;5(12):e1000792 10.1371/journal.pgen.1000792 20041220 PMC2791168

[15] Wang X, Lin Y, Song C, Sibille E, Tseng GC. Detecting disease-associated genes with confounding variable adjustment and the impact on genomic meta-analysis: with application to major depressive disorder. BMC bioinformatics. 2012;13:52 10.1186/1471-2105-13-52 22458711 PMC3342232

[16] Franceschini A, Szklarczyk D, Frankild S, Kuhn M, Simonovic M, Roth A, et al STRING v9.1: protein-protein interaction networks, with increased coverage and integration. Nucleic Acids Res. 2013;41(Database issue):D808–15. 10.1093/nar/gks1094 23203871 PMC3531103

[17] Teixeira VH, Olaso R, Martin-Magniette ML, Lasbleiz S, Jacq L, Oliveira CR, et al Transcriptome analysis describing new immunity and defense genes in peripheral blood mononuclear cells of rheumatoid arthritis patients. PloS One. 2009;4(8):e6803 10.1371/journal.pone.0006803 19710928 PMC2729373

[18] Barnes MG, Aronow BJ, Luyrink LK, Moroldo MB, Pavlidis P, Passo MH, et al Gene expression in juvenile arthritis and spondyloarthropathy: pro-angiogenic ELR+ chemokine genes relate to course of arthritis. Rheumatology. 2004;43(8):973–9. 15150433 10.1093/rheumatology/keh224

[19] Lee HM, Mima T, Sugino H, Aoki C, Adachi Y, Yoshio-Hoshino N, et al Interactions among type I and type II interferon, tumor necrosis factor, and beta-estradiol in the regulation of immune response-related gene expressions in systemic lupus erythematosus. Arthritis Res Ther. 2009;11(1):R1 10.1186/ar2584 19121222 PMC2688231

[20] Lee HM, Sugino H, Aoki C, Nishimoto N. Underexpression of mitochondrial-DNA encoded ATP synthesis-related genes and DNA repair genes in systemic lupus erythematosus. Arthritis Res Ther. 2011;13(2):R63 10.1186/ar3317 21496236 PMC3132058

[21] Ramos YF, Bos SD, Lakenberg N, Bohringer S, den Hollander WJ, Kloppenburg M, et al Genes expressed in blood link osteoarthritis with apoptotic pathways. Ann Rheum Dis. 2014;73(10):1844–53. 10.1136/annrheumdis-2013-203405 23864235

[22] Pimentel-Santos FM, Ligeiro D, Matos M, Mourao AF, Costa J, Santos H, et al Whole blood transcriptional profiling in ankylosing spondylitis identifies novel candidate genes that might contribute to the inflammatory and tissue-destructive disease aspects. Arthritis Res Ther. 2011;13(2):R57 10.1186/ar3309 21470430 PMC3132052

[23] Zhernakova A, van Diemen CC, Wijmenga C. Detecting shared pathogenesis from the shared genetics of immune-related diseases [review]. Nat Rev Genet. 2009;10(1):43–55. 10.1038/nrg2489 19092835

[24] Audo R, Calmon-Hamaty F, Baeten D, Bruyer A, Combe B, Hahne M, et al Mechanisms and clinical relevance of TRAIL-triggered responses in the synovial fibroblasts of patients with rheumatoid arthritis. Arthritis Rheum. 2011;63(4):904–13. 10.1002/art.30181 21305500

[25] Lub-de Hooge MN, de Vries EG, de Jong S, Bijl M. Soluble TRAIL concentrations are raised in patients with systemic lupus erythematosus. Ann Rheum Dis. 2005;64(6):854–8. 15564310 10.1136/ard.2004.029058 PMC1755511

[26] Zai-Xing Y, Yan L, Hao W, Ye Z, Chang L, Ren-Qian Z. Preliminary clinical measurement of the expression of TNF-related apoptosis inducing ligand in patients with ankylosing spondylitis. J Clin Lab Anal. 2008;22(2):138–45. 10.1002/jcla.20231 18348311 PMC6649006

[27] Lee SW, Lee HJ, Chung WT, Choi SM, Rhyu SH, Kim DK, et al TRAIL induces apoptosis of chondrocytes and influences the pathogenesis of experimentally induced rat osteoarthritis. Arthritis Rheum. 2004;50(2):534–42. 14872496 10.1002/art.20052

[28] Guo P, Zhang Q, Zhu Z, Huang Z, Li K. Mining gene expression data of multiple sclerosis. PloS One. 2014;9(6):e100052 10.1371/journal.pone.0100052 24932510 PMC4059716

[29] Pingiotti E, Cipriani P, Marrelli A, Liakouli V, Fratini S, Penco M, et al Surface expression of fractalkine receptor (CX3CR1) on CD4+/CD28 T cells in RA patients and correlation with atherosclerotic damage. Ann N Y Acad Sci. 2007;1107:32–41. 17804530 10.1196/annals.1381.004

[30] Broux B, Pannemans K, Zhang X, Markovic-Plese S, Broekmans T, Eijnde BO, et al CX(3)CR1 drives cytotoxic CD4(+)CD28(-) T cells into the brain of multiple sclerosis patients. J Autoimmun. 2012;38(1):10–9. 22123179 10.1016/j.jaut.2011.11.006

[31] Astorri E, Scrivo R, Bombardieri M, Picarelli G, Pecorella I, Porzia A, et al CX3CL1 and CX3CR1 expression in tertiary lymphoid structures in salivary gland infiltrates: fractalkine contribution to lymphoid neogenesis in Sjogren's syndrome. Rheumatology. 2014;53(4):611–20. 10.1093/rheumatology/ket401 24324211

[32] Han KH, Ryu JW, Lim KE, Lee SH, Kim Y, Hwang CS, et al Vascular expression of the chemokine CX3CL1 promotes osteoclast recruitment and exacerbates bone resorption in an irradiated murine model. Bone. 2014;61:91–101. 10.1016/j.bone.2013.12.032 24401612

[33] Assassi S, Reveille JD, Arnett FC, Weisman MH, Ward MM, Agarwal SK, et al Whole-blood gene expression profiling in ankylosing spondylitis shows upregulation of toll-like receptor 4 and 5. J Rheumatol. 2011;38(1):87–98. 10.3899/jrheum.100469 20952467 PMC3014385

[34] Chamberlain ND, Vila OM, Volin MV, Volkov S, Pope RM, Swedler W, et al TLR5, a novel and unidentified inflammatory mediator in rheumatoid arthritis that correlates with disease activity score and joint TNF-alpha levels. J Immunol. 2012;189(1):475–83. 10.4049/jimmunol.1102977 22661088 PMC3614068

[35] Sanchez E, Callejas-Rubio JL, Sabio JM, Gonzalez-Gay MA, Jimenez-Alonso J, Mico L, et al Investigation of TLR5 and TLR7 as candidate genes for susceptibility to systemic lupus erythematosus. Clin Exp Rheumatol. 2009;27(2):267–71. 19473567

[36] Balada E, Castro-Marrero J, Felip L, Ordi-Ros J, Vilardell-Tarres M. Clinical and serological findings associated with the expression of ITGAL, PRF1, and CD70 in systemic lupus erythematosus. Clin Exp Rheumatol. 2014;32(1):113–6. 24238281

[37] Romero V, Fert-Bober J, Nigrovic PA, Darrah E, Haque UJ, Lee DM, et al Immune-mediated pore-forming pathways induce cellular hypercitrullination and generate citrullinated autoantigens in rheumatoid arthritis.Sci Transl Med. 2013;5(209):209ra150 10.1126/scitranslmed.3006869 24174326 PMC4032227

[38] Raffeiner B, Dejaco C, Duftner C, Kullich W, Goldberger C, Vega SC, et al Between adaptive and innate immunity: TLR4-mediated perforin production by CD28null T-helper cells in ankylosing spondylitis. Arthritis Res Ther. 2005;7(6):R1412–20. 16277694 10.1186/ar1840 PMC1297589

[39] Li H, Wan A. Apoptosis of rheumatoid arthritis fibroblast-like synoviocytes: possible roles of nitric oxide and the thioredoxin 1. Mediators Inflamm. 2013;2013:953462 10.1155/2013/953462 23690674 PMC3649754

[40] Takata Y, Hamada D, Miyatake K, Nakano S, Shinomiya F, Scafe CR, et al Genetic association between the PRKCH gene encoding protein kinase Ceta isozyme and rheumatoid arthritis in the Japanese population. Arthritis Rheum. 2007;56(1):30–42. 17195206 10.1002/art.22262

[41] Sugihara M, Tsutsumi A, Suzuki E, Wakamatsu E, Suzuki T, Ogishima H, et al Effects of infliximab therapy on gene expression levels of tumor necrosis factor alpha, tristetraprolin, T cell intracellular antigen 1, and Hu antigen R in patients with rheumatoid arthritis. Arthritis Rheum. 2007;56(7):2160–9. 17599736 10.1002/art.22724

[42] Dziarski R, Wang Q, Miyake K, Kirschning CJ, Gupta D. MD-2 enables Toll-like receptor 2 (TLR2)-mediated responses to lipopolysaccharide and enhances TLR2-mediated responses to Gram-positive and Gram-negative bacteria and their cell wall components. J Immunol. 2001 2 1;166(3):1938–44. 11160242 10.4049/jimmunol.166.3.1938

[43] Heng TS, Painter MW, Immunological Genome Project C. The Immunological Genome Project: networks of gene expression in immune cells. NatImmunol. 2008;9(10):1091–4. 10.1038/ni1008-1091 18800157

